# Influence of Ether—A Suit against a Dental Surgeon

**Published:** 1855-01

**Authors:** 


**Verdict against Dr. S. T. Beale for Rape, while the Defendant was under the influence of Ether.**

This is one of the most unaccountable verdicts we ever heard of. A young woman swears to being forced while under the influence of ether, by a man whose character stands as high as that of any other in this community. The defence was neglected, and a new trial is demanded by an indignant community.—*Phila. Med. and Sur. Journal.*

The above case involves a medico-legal question of some importance. Was the lady in question under the full influence of the anæsthetic at the time of the perpetration of the outrage which she charges against the defendant? The chief purpose and object in the exhibition of an anæsthetic of any kind, is to destroy sensibility so that the patient may not be conscious of the suffering incident to the performance of a painful operation. The only other benefit to be derived from it would be to quiet muscular irritability, and consequent contractibility, so that the operator may not be interrupted by the movements of the patient. But the benefits of anæsthesia do not rest upon this, which is a minor consideration when compared with the humane aspects of the question. Those who herald forth the great advantages of etherization, place it upon the ground that it renders the individual to whom it becomes necessary to exhibit it, insensible to the pain and agony consequent upon important operations, or other conditions of great suffering. This is the experience of every surgeon, for if the patient comes promptly and fully under the influence of ether or chloroform, and the operation is completed before he awakes to consciousness, and then questioned on the subject, his replies will show that he knew nothing of it.

Now this is the great desideratum claimed for the exhibition, and the use of the anæsthetic, and it is the only one which would justify the exhibition of so subtle and dangerous a remedy by inhalation. Now if this young lady was under the use of the chloroform, was she susceptible of feeling and consciousness, without the power of resistance? This brings us to another enquiry, namely: will it control the motor nerves, and the nerves of sensibility still remain alive to impressions. Experience and observation shows that persons under its influence do often make movements and yet be unconscious of impressions, or when free from its influence retain no remembrance of any circumstance which had transpired, even when highly sensitive tissues had been invaded by the knife.

It is our conviction, that if she were fully under the influence of the chloroform, so as to deprive her of all power of resistance, she was wholly unconscious of the act, and could have had no recollection of it.

This circumstance occurred about the time of our recent visit to Philadelphia, and was discussed freely in certain circles. The reputation of the Surgeon Dentist was said to be above reproach, and he had an interesting family at the time. It appears that after all this should have transpired, she took his arm while he conducted her to her carriage, and during this time laughed and smiled in his face *very endearingly*, and continued a pleasant *tete a tete* with him at the carriage after she had taken her seat.

If any wrong were perpetrated and these facts last stated are true, then for *his* purposes we think the anæsthetic to have been unnecessary.

Now we will admit that if he did, as charged, take an advantage of her somnolent condition and perpetrate such an outrage upon her person, he deserves a punishment the very highest known to the law. We can find no palliation for such an offence against the innocence and purity of a defenceless female. We can scarcely conceive of a higher crime, and the law should make a conspicuous example of *him* who will dare to violate the sacred trust reposed in him.

But we are incredulous as to his guilt and will continue to be until we see stronger proofs than have been presented. We must know that there were other evidences of the *rape* than her testimony alone. Justice claims that he should have another hearing, and we hope that this new point in legal medicine may be settled by the testimony of competent scientific witnesses.

As we have before stated Dr. Beale sustains an unblemished reputation in Philadelphia, and the announcement of his guilt and that the charges were made by a young lady of good standing in society, were circumstances which awakened much surprise and astonishment on the part of the citizens and much speculation was indulged in.

This case has its warning however. The charlatan with this subtle agent in his pocket, would have it in his power to perpetrate outrages and the victim at the time be insensible and unconscious of it. The retired situation of dental rooms, and other circumstances arising out of the discharge of his duty in his profession would favor the schemes of a villian in the perpetration of acts of the foulest atrocity upon unprotected and confiding females.

